# Machine Learning for Histologic Subtype Classification of Non-Small Cell Lung Cancer: A Retrospective Multicenter Radiomics Study

**DOI:** 10.3389/fonc.2020.608598

**Published:** 2021-01-14

**Authors:** Fengchang Yang, Wei Chen, Haifeng Wei, Xianru Zhang, Shuanghu Yuan, Xu Qiao, Yen-Wei Chen

**Affiliations:** ^1^ Department of Radiology, Shandong Cancer Hospital and Institute, Cheeloo College of Medicine, Shandong University, Jinan, China; ^2^ Department of Implantology, School and Hospital of Stomatology, Cheeloo College of Medicine, Shandong University, Jinan, China; ^3^ First Clinical Medical College, Shandong University of Traditional Chinese Medicine, Jinan, China; ^4^ School of Control Science and Engineering, Shandong University, Jinan, China; ^5^ Department of Radiation Oncology, Shandong Cancer Hospital and Institute, Shandong First Medical University and Shandong Academy of Medical Sciences, Jinan, China; ^6^ Graduate School of Information Science and Engineering, Ritsumeikan University, Shiga, Japan; ^7^ Research Center for Healthcare Data Science, Zhejiang Lab, Hangzhou, China

**Keywords:** non-small cell lung cancer, radiomics, machine learning, feature selection, classification

## Abstract

**Background:**

Histologic phenotype identification of Non-Small Cell Lung Cancer (NSCLC) is essential for treatment planning and prognostic prediction. The prediction model based on radiomics analysis has the potential to quantify tumor phenotypic characteristics non-invasively. However, most existing studies focus on relatively small datasets, which limits the performance and potential clinical applicability of their constructed models.

**Methods:**

To fully explore the impact of different datasets on radiomics studies related to the classification of histological subtypes of NSCLC, we retrospectively collected three datasets from multi-centers and then performed extensive analysis. Each of the three datasets was used as the training dataset separately to build a model and was validated on the remaining two datasets. A model was then developed by merging all the datasets into a large dataset, which was randomly split into a training dataset and a testing dataset. For each model, a total of 788 radiomic features were extracted from the segmented tumor volumes. Then three widely used features selection methods, including minimum Redundancy Maximum Relevance Feature Selection (mRMR), Sequential Forward Selection (SFS), and Least Absolute Shrinkage and Selection Operator (LASSO) were used to select the most important features. Finally, three classification methods, including Logistics Regression (LR), Support Vector Machines (SVM), and Random Forest (RF) were independently evaluated on the selected features to investigate the prediction ability of the radiomics models.

**Results:**

When using a single dataset for modeling, the results on the testing set were poor, with AUC values ranging from 0.54 to 0.64. When the merged dataset was used for modeling, the average AUC value in the testing set was 0.78, showing relatively good predictive performance.

**Conclusions:**

Models based on radiomics analysis have the potential to classify NSCLC subtypes, but their generalization capabilities should be carefully considered.

## Introduction

Lung cancer is the leading cause of cancer death in many countries (1, 2). Especially in China, lung cancer is the most common cancer with more than 430,000 deaths per year (3). According to the characteristics of cancer cells under the microscope, lung cancer is broadly classified into two types: small cell lung cancer (SCLC) and non-small cell lung cancers (NSCLC). NSCLC is the most common type of lung cancer, accounting for about 80% of all lung cancers. Squamous cell carcinoma (SCC) and adenocarcinoma (ADC) are the most common histological subtypes of NSCLC. The classification criteria is based on the histologic features, i.e., ADC appears as carcinoma of acinar/tubular structure or mucin production, while SCC appears as carcinoma with keratinization or intercellular bridges (4).

Since the treatment methods of SCC and ADC are quite different, it is of great significance to accurately distinguish SCC from ADC in patients with lung cancer (5, 6). For instance, pemetrexed (a multiple-enzyme inhibitor) has greater efficacy in ADC patients than in SCC patients (7). Pathological diagnosis is commonly regarded as the gold standard for distinguishing ADC from SCC. However, it is invasive and requires needle biopsy or surgery. The tumor may be heterogeneous, which may lead to sampling errors and affect biopsy results. In addition, the risk of complications is also an important factor that must be considered. These limitations of pathological diagnosis prompt us to develop non-invasive and accurate alternative methods.

Radiomics refers to extracting high-throughput features from medical images and mining the potential relationships between quantitative image features and pathophysiology characteristics. Radiomics analysis can be used for predicting various clinical outcomes, such as survival, distant metastases, and molecular characteristics classification (8–10). Several studies have focused on the identification of histologic subtype of NSCLC based on radiomics. Wu et al. (11) constructed two study cohorts with 350 patients and extracted 440 radiomic features for each sample. They applied 24 feature selection methods and 3 classification methods to identify SCC and ADC, of which the Naive Bayes method achieved the highest AUC of 0.72. Zhu et al. (12) retrospectively studied 129 patients with NSCLC and extracted 485 features from manually labeled tumor regions. Five features were selected to construct a radiomics signature by using a logistic regression method. This radiomic signature achieved an AUC of 0.893 in the test set. Chaunzwa et al. (13) retrospectively studied 157 patients with NSCLC to classify ADC or SCC. They used a VGG-16 neural network to extract deep features from CT images and classify them with fully connected layers. Besides, they also independently evaluated the extracted features using three machine learning classification models. The results showed that all models were able to classify tumor histology, of which the neural network achieved the highest performance with an AUC of 0.751.

Although these studies have achieved excellent results, there are still some critical problems that need to be solved: 1) Many radiomics studies generally have small size datasets, thus limiting the performance and the potential clinical applicability of these models. 2) The research methods are relatively simple, and there are few methods of feature selection and classifier for comparison. The differences between different research methods are not fully discussed, which reduces the credibility of the model and limits the application of the models in the clinic.

In order to solve the above problems, we collected three datasets from different centers. Each dataset was used as the training set to build a model and tested in the remaining two datasets. Then, we combined all the datasets into one large dataset to build a model; this dataset was randomly divided into a training set and a testing set. For each dataset, a total of 788 radiomic features were extracted from the segmented tumor volumes of corresponding CT images. Three widely used features selection methods, minimum Redundancy Maximum Relevance Feature Selection (mRMR), Sequential Forward Selection (SFS), Least Absolute Shrinkage and Selection Operator (Lasso) were used to select the most important features. Three widely used classification models were independently evaluated on the selected features: Logistics Regression (LR), Support Vector Machines (SVM), and Random Forest (RF). We aim to build models through multi-center datasets to thoroughly study the potential of radiomics in identifying SCC and ADC.

## Materials and Methods

**Figure 1:**
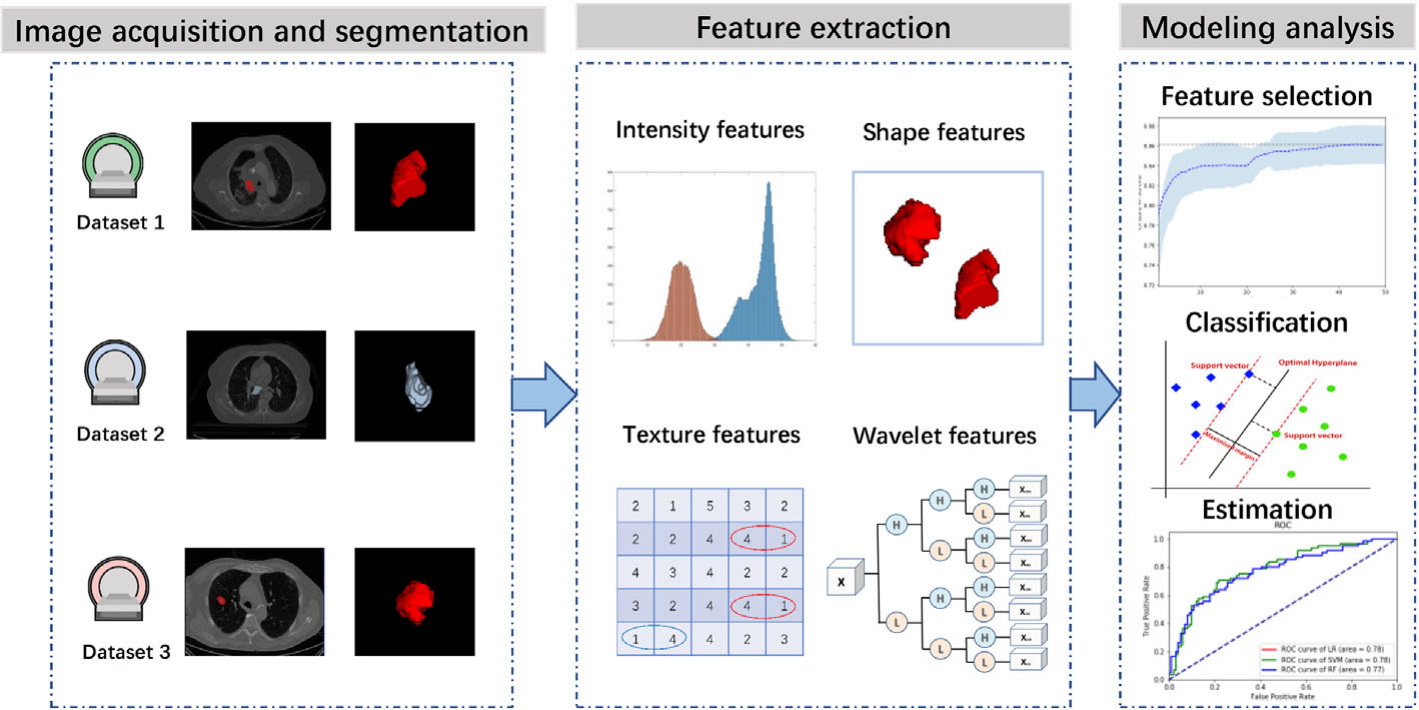
Workflow of this study.

**Figure 1** presents the workflow of this study, including image acquisition and segmentation, feature extraction, feature selection, classifier construction and evaluation. In the following sections, we will describe these steps in detail.

### Datasets

We utilized 3 independent datasets in this study that were collected from China institution and open-access online repositories.

#### Dataset 1

This retrospective study has been approved by our institutional review board and does not require patient informed consent. From June 2014 to June 2019, 324 patients with a diagnosis of lung cancer were retrospectively collected. The inclusion criteria are as follows: (1) pathologically confirmed lung cancer; (2) CT images can be obtained before treatment. Exclusion criteria were as follows: (1) small cell lung cancer (n = 3); (3) grade of preoperative biopsy evidence was not available (n = 17). Finally, 302 patients were selected for this study. Tumors were classified into ADC or SCC based on pathological information. All pulmonary CT examinations were performed using four CT scanners, with tube voltage of 120 kVp, tube current of 220 mAs, and inter-layer slice thickness of 4–5 mm. For each patient, the tumor region was contoured in a slice-by-slice manner on CT images by an experienced radiologist (with eight years of experience) using Medical Imaging Interaction Toolkit (MITK) software (14) (version 2013.12.0; http://www.mitk.org/), and then confirmed by another experienced radiologist (with 15 years of experience). The final consensus was reached by group discussion if there were discrepant interpretations.

#### Dataset 2

This dataset was obtained from The Cancer Imaging Archive (TCIA) (15) and included 422 patients with NSCLC treated at Maastricht University Medical Center (16). All patients underwent a CT scan. Depending on the patient’sbody type, the scanning scheme was slightly different. The tube voltage was 120–140 kVp and the tube current was 40–80 mAs. The reconstructed pixel size was 0.977×977mm, the matrix size was 512×512, and the layer thickness between slices was 3 mm. For all CT images, the doctor performed manual tumor region segmentation. From all the samples, 203 samples that meet the requirements of this study were selected.

#### Dataset 3

This dataset was obtained from TCIA (15) and included 211 NSCLC patients (17). This is a retrospectively collected dataset through different CT equipments and different imaging parameters, with tube voltage of 80–140 kVp, tube current of 124–699 mAs, and inter-layer slice thickness of 0.625–3 mm. For all CT images, an undisclosed automatic segmentation algorithm was used for segmentation and then manually adjusted by the doctors. From all the samples, 140 samples that meet the requirements of this study were selected.

**Figure 2:**
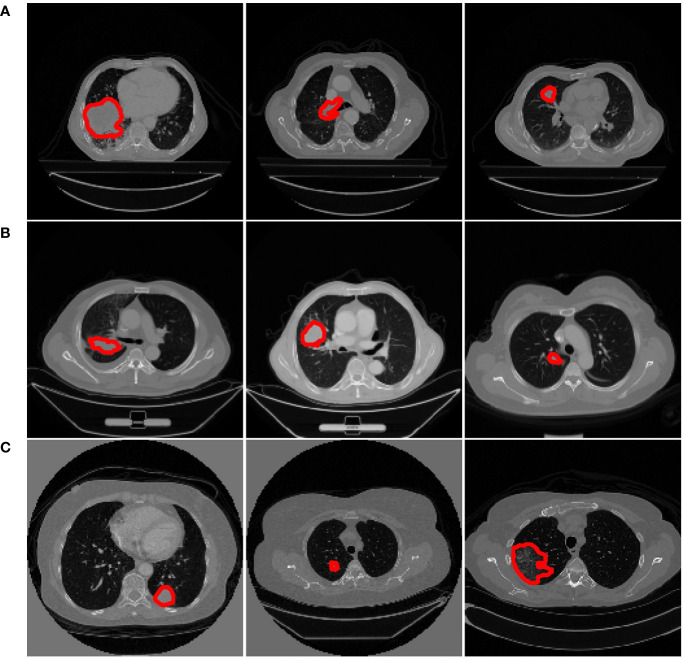
Examples from different datasets. Each row represents three axial slices of different datasets; **(A)** Dataset 1. **(B)** Dataset 2. **(C)** Dataset 3. A red contour that outlined by the physician is displayed to identify the cancer area in each patient scan.

Some slices from the above datasets are displayed in **Figure 2** to show the variety in cancer locations, shapes, and appearances of the different datasets.

### Feature Extraction and Selection

Before feature extraction, we resampled all the CT images to a 1×1×1 *mm* ^3^ voxel size. According to the radiomic features described by Imaging Biomarker Standardization Initiative (IBSI), a wide range of features including intensity features, shape features, texture features, and wavelet features were extracted from the segmented cancer regions (18).

Intensity features use first-order statistics (energy, entropy, standard deviation, skewness, kurtosis, etc.) to quantify the tumor intensity feature, which are calculated from the histogram of all tumor voxel intensity values. Shape features describe the shape of the tumor, such as sphericity or compactness of the tumor. Texture features can quantify intratumor heterogeneity differences in the texture that is observable within the tumor volume. These features are calculated in all three-dimensional directions within the tumor volume, thereby taking the spatial location of each voxel compared with the surrounding voxels into account. Texture features quantify the intratumor heterogeneity by using the Gray Level Cooccurrence (GLCM), Gray Level Run Length Matrices (GLRLM), Gray Level Size Zone Matrix (GLSZM), Neighbouring Gray Tone Difference Matrix (NGTDM) and Gray Level Dependence Matrix (GLDM). Wavelet features calculate the intensity and textural features from wavelet decompositions of the original image, thereby focusing the features on different frequency ranges within the tumor volume. All feature extraction algorithms were implemented in Pyradiomics toolkit (19).

To eliminate the differences in the value scales of the radiomic features, feature normalization was performed before feature selection. For features in the training group, each feature for a specific patient was subtracted by the mean value and divided by standard deviation value from this group. The same normalization method was applied to features in the validation group using the mean values and standard deviation values calculated based on the training group.

Too many features will increase the computational cost, and the redundancy between features will reduce the accuracy of the classification. Furthermore, the number of features is more than the number of samples in this work, which will increase the probability of overfitting. Therefore, feature selection is essential. There are three main types of feature selection algorithms: filter methods, wrapper methods, and embedded methods. Based on previous works, we selected three widely used feature selection methods, namely: minimum redundancy maximum correlation method (mRMR) (20), sequential forward selection method (SFS) (21), and least absolute shrinkage and selection operator (Lasso) (22).

mRMR is a multivariate filtering feature selection algorithm, which finds the optimal subset of features by considering both the importance of features and the correlation between them, that is, maximizing the correlation between features and categorical variables, while minimizing the redundancy between features. In the set S with N features, the correlation D of the features is calculated as follows: 

(1) $$D=\frac{1}{\left|S\right|}\underset{x_{i}\in S}{\sum }I\left(x_{i};c\right)$$

Where *I* represents mutual information and the redundancy between features is expressed as: 

(2) $$R=\frac{1}{|S{|}^{2}}\underset{x_{i},x_{j}\in S}{\sum }I\left(x_{i},x_{j}\right)$$

The goal of mRMR is to find the feature subset where *D* – *R* takes the maximum value. SFS is a kind of wrapper method that uses a bottom-up search strategy that starts from an empty feature set and gradually adds features selected by evaluation function. At each iteration, the feature to be added is selected from the remaining available features that have not been added to the feature set. Then, the final selected features should produce the best classification performance compared with the any other feature set (23). Lasso is a kind of embedded method that is widely used in radiomic feature selection of high dimensional data with relatively small sample size. It is based on ℓ_1_-norm of the coefficient of a linear classifier. Some of the coefficients of the learned classifier may equal zero. Since each coefficient is associated with a feature, so feature selection is achieved by retaining features with non-zero coefficients.

### Classifiers Construction

We evaluated three classification algorithms: logistic regression (LR), support vector machine (SVM), and random forest (RF). LR is a classical machine learning algorithm that was usually used for binary classification tasks. The model attempts to estimate the probability *p*(*y* = 1|*x*), that is, the probability of a positive result *y* = 1 under the given data *x*. The advantage of logistic regression is that the training speed is fast, and discrete and continuous variables can be used as inputs. The disadvantage is that it is a linear model, and the classification effect is not good enough in the face of complex data problems, but the logistic regression model can achieve good results on many datasets, and it is easy to implement and can be used as a basic modeling method (24). SVM is another widely used classification algorithm that attempts to separate the data by computing the decision boundary. This decision boundary, also called the hyperplane, is orientated in such a way that it is as far away as possible from the closest data points (support vectors) from each class (25). SVM is a powerful method for obtaining good classification results by using only a few data points (26). RF is an ensemble learning method, known for its high performance and generalizability. It uses bootstrap resampling to extract multiple samples from the original sample, and constructs a decision tree for each bootstrap sample, and then combines these decision trees together to obtain the final classification (27).

### Statistical Analysis

The statistical analyses were performed with R 3.1.2 (http://www.R-project.org) and Python (version 3.6.4) machine learning library scikit-learn (version 0.19.1). Univariate analysis for clinical data was performed by using the Chi-square (χ^2^) test or Mann-Whitney U test, as appropriate. The categorical variable (such as gender and category probability) was analyzed using the χ^2^ test. The continuous variable (such as age) was analyzed using the Mann-Whitney U test.

## Results

### Patients Statistics

**Table 1** lists the clinical data of the patients in the three datasets. The percentages of SCC in dataset 1, 2, and 3 were 29, 75, and 20%, respectively. Among them, the category probability between dataset 1 and dataset 2 is statistically different (*P* < 0.001, χ^2^ test); the category probability between dataset 1 and dataset 3 is statistically different (*P* = 0.04959, χ^2^ test); the category probability between dataset 2 and dataset 3 is statistically different (*P* < 0.001, χ^2^ test).

**Table 1:** Patients statistics.

	Dataset 1		Dataset 2		Dataset 3	
	ADC (n = 215)	SCC (n = 87)	ADC (n = 51)	SCC (n = 152)	ADC (n = 112)	SCC (n = 28)
**age**						
range (median)	36–89 (59)	32–81 (65)	45–85 (68)	33–88 (70)	43–87 (68)	57–83 (71)
mead+std	59±10	64±9	67±9	70±10	68±9	71±6
**sex**						
male	108	79	32	112	80	24
female	107	8	19	40	32	4

The percentages of males in dataset 1, 2, and 3 are 62, 71, and 74%, respectively. Among them, the probability of gender in dataset 1 is statistically significant difference (*P* < 0.001, χ^2^ test); the probability of gender in dataset 2 is not statistically significant difference (*P* = 0.01365, χ^2^ test); the probability of gender in dataset 3 is not statistically significant difference (*P* = 0.01219, χ^2^ test).

There is a statistically significant difference between the age of SCC and ADC in dataset 1 (*P* < 0.001, Mann-Whitney U test); there is no statistical difference between the age of SCC and ADC in dataset 2 (*P* = 0.06693, Mann -Whitney U test); there is no statistical difference between the ages of SCC and ADC in dataset 3 (*P* = 0.1501, Mann-Whitney U test). As can be seen from the above results, there exist significant differences between different datasets.

### Feature Extraction and Selection Results

**Figure 3:**
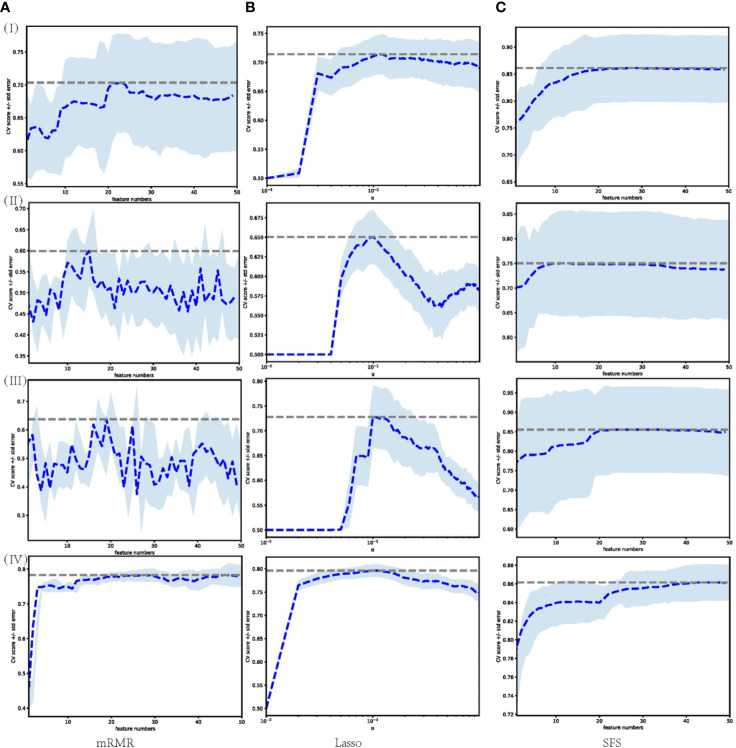
Feature selection process performed on different datasets through different feature selection methods. Each row represents different datasets; (I) Dataset 1. (II) Dataset 2. (III) Dataset 3. (IV) Merged dataset. Each column represents feature selection methods; **(A)** mRMR. **(B)** Lasso. **(C)** SFS.

A large number of features were extracted from the tumor volume, where each sample contains 788 features. To select the most distinguishing feature subset, we applied three widely used feature selection methods. For each method, we applied Grid search and 5-fold cross-validation to select the best hyper-parameters. The feature selection process is shown in **Figure 3**, where (I)–(IV) represent the feature selection process based on dataset 1, dataset 2, dataset 3, and merged dataset, respectively. The blue dashed line represents the average AUC value of the 5-fold cross-validation, and the shading represents the standard deviation. For mRMR and SFS, the hyperparameter is the number of features, and for Lasso, the hyperparameter is the regularization parameter α. Using the AUC value as the criterion for hyperparameter selection, it can be seen from the figure that for models 1–3, the AUC value is relatively low, and the standard deviation is larger, the Lasso feature selection method shows the best stability.

**Table 2** lists the number of features selected by the three feature selection methods in the four training sets and the jointly selected features. For the model trained in dataset 1, mRMR, SFS, and Lasso selected 24, 28, and 28 features, respectively, and 1 feature was jointly selected. It is wavelet-LLH firstorder_Skewness, which measures the asymmetry of value distribution of the tumor area under wavelet transform. For the model trained in dataset 2, mRMR, SFS, and Lasso selected 17, 13, and 9 features, respectively, and there were no features that were jointly selected. For the model trained in dataset 3, mRMR, SFS, and Lasso selected 17, 24, and 7 features, respectively, and there were no features that were jointly selected. For the model trained in the merged dataset, mRMR, SFS, and Lasso selected 29, 46, and 29 features, respectively, and 1 feature was jointly selected, which is wavelet-HHL_glcm_ClusterShade, that measures the skewness of GLCM features under wavelet transform. A higher ClusterShade indicates greater asymmetry. According to the above results, it can be seen that different feature selection methods select different features, and there is a great inconsistency between the selected features.

**Table 2:** Commonly selected features.

	mRMR	SFS	Lasso	Common features
dataset 1	24	28	28	wavelet-LLH_firstorder_Skewness
dataset 2	17	13	9	None
dataset 3	17	24	7	None
merged dataset	29	46	29	wavelet-HHL_glcm_ClusterShade

### Classification Result

**Figure 4:**
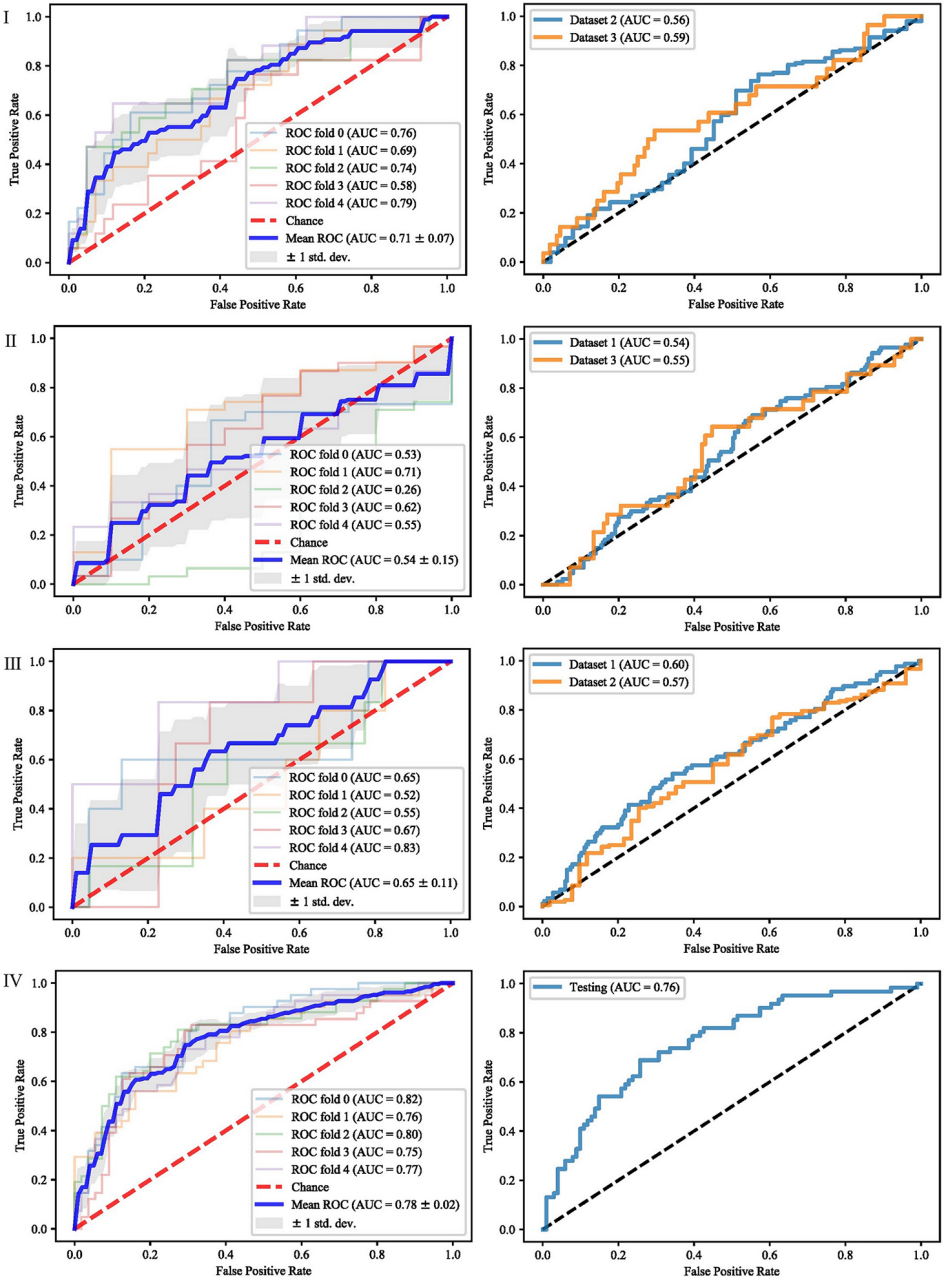
ROCs of different datasets achieved through mRMR and SVM. Each row represents different datasets; (I) Dataset 1. (II) Dataset 2. (III) Dataset 3. (IV) Merged dataset.

**Figure 5:**
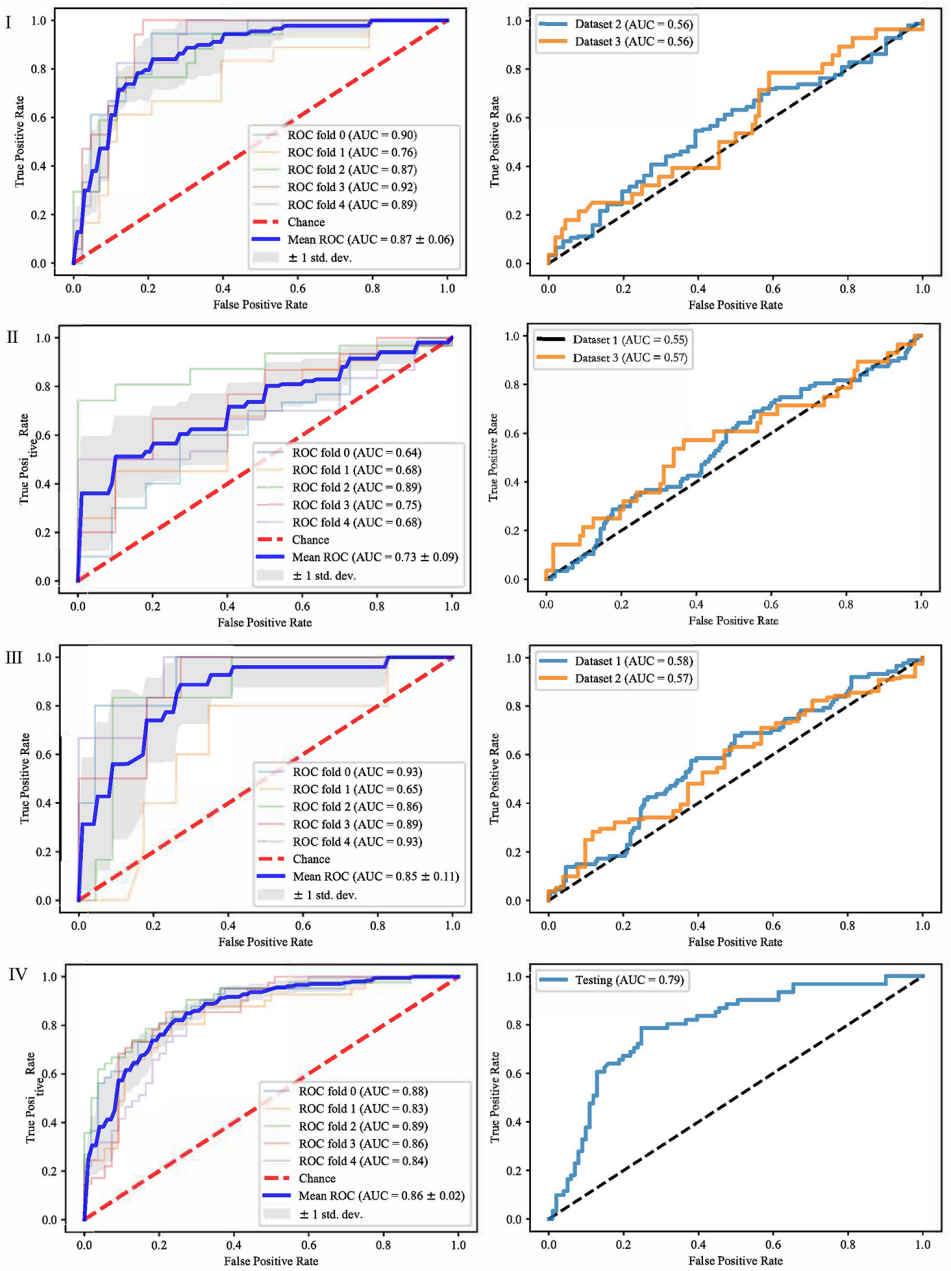
ROCs of different datasets achieved through SFS and SVM. Each row represents different datasets; (I) Dataset 1. (II) Dataset 2. (III) Dataset 3. (IV) Merged dataset.

**Figure 6:**
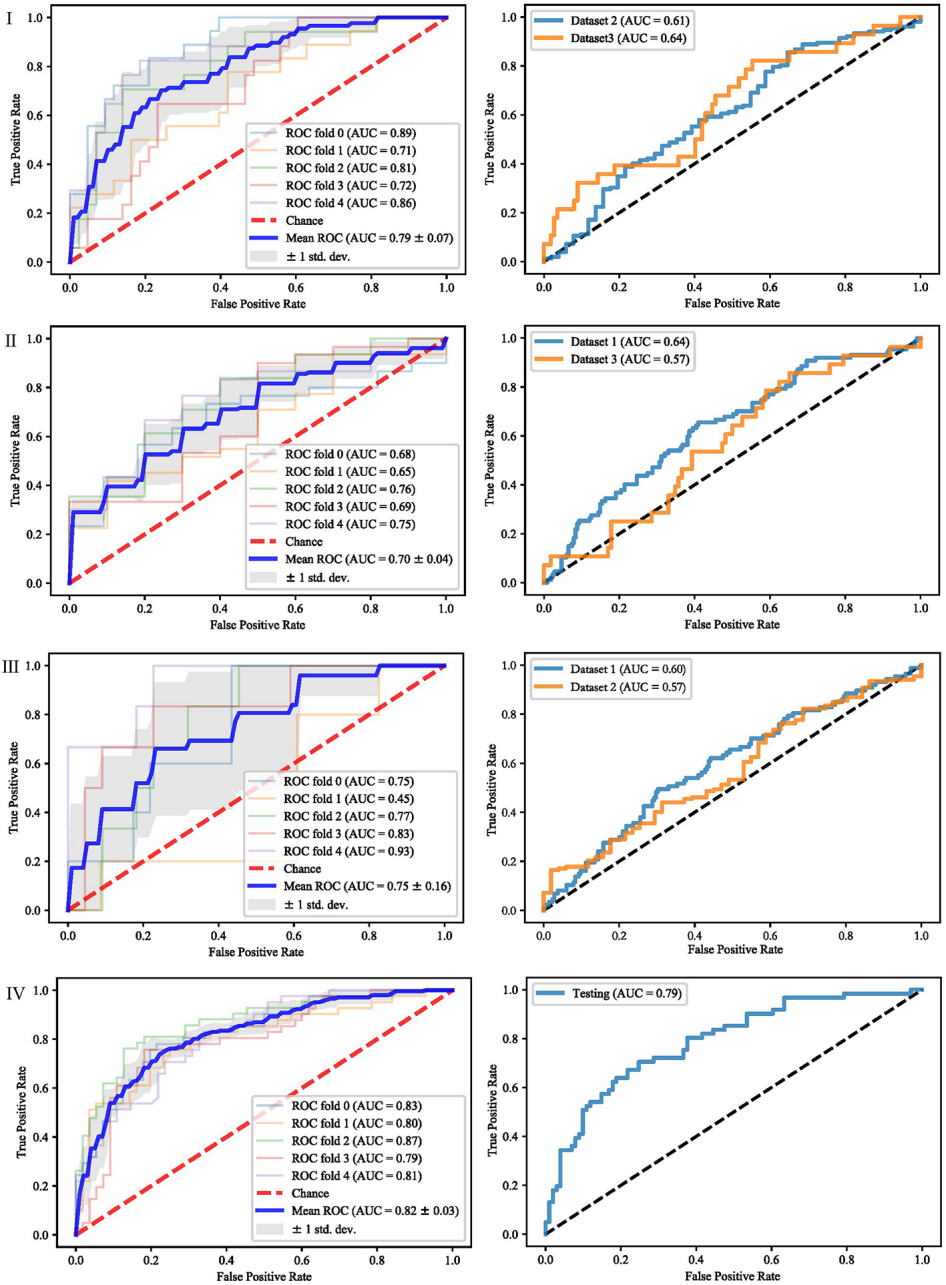
ROCs of different datasets achieved through Lasso and SVM. Each row represents different datasets; (I) Dataset 1. (II) Dataset 2. (III) Dataset 3. (IV) Merged dataset.

It can be seen from the previous section that different feature selection methods selected distinct feature subsets. To evaluate these feature subsets, we used three classifiers for modeling and analysis. Taking the SVM method as an example, **Figures 4**–**6** show the ROC curves of different datasets that obtained by three feature selection methods. The left column of each figure shows the 5-fold cross-validated ROC curves of the training dataset, and the right column shows the ROC curves of the testing dataset.

When dataset 1 was used to build the model and mRMR method was used to select features, the average AUC value of 5-fold cross validation was 0.71, and the AUC value in dataset 2 and 3 were 0.56 and 0.59, respectively. When SFS feature selection method was used, the average AUC value of 5-fold cross validation was 0.87, and the AUC value in dataset 2 and dataset 3 were 0.56 and 0.56, respectively. When Lasso feature selection method was used, the average AUC value of 5-fold cross validation was 0.79, and the AUC values in dataset 2 and dataset 3 were 0.61 and 0.64, respectively.

When dataset 2 was used to build the model and mRMR method was used to select features, the average AUC value of 5-fold cross-validation was 0.54, and the AUC values in dataset 1 and dataset 3 were 0.54 and 0.55, respectively. When SFS feature selection method was used, the average AUC value of 5-fold cross-validation was 0.73, and the AUC values in dataset 1 and dataset 3 were 0.55 and 0.57, respectively. When Lasso feature selection method was used, the average AUC value of 5-fold cross-validation was 0.70, and the AUC values in dataset 1 and dataset 3 were 0.64 and 0.57, respectively.

When dataset 3 was used to build the model and mRMR method was used to select features, the average AUC value of 5-fold cross-validation was 0.65, and the AUC values in dataset 1 and dataset 3 were 0.60 and 0.57, respectively. When SFS feature selection method was used, the average AUC value of 5-fold cross-validation was 0.85, and the AUC values in dataset 1 and dataset 3 were 0.58 and 0.57, respectively. When the Lasso feature selection method was used, the average AUC value of 5-fold cross-validation was 0.75, and the AUC values in dataset 1 and dataset 3 were 0.60 and 0.57, respectively.

When the merged dataset was used to build the model and mRMR method was used to select features, the average AUC value of 5-fold cross-validation was 0.78, and the AUC value in the testing set was 0.76. When the SFS feature selection method was used, the average AUC of 5-fold cross-validation was 0.86, and the AUC value in the test set was 0.79. When the Lasso feature selection method was used, the average AUC value of 5-fold cross-validation was 0.82, and the AUC value in the test set was 0.79.

It can be seen from the above results that although different feature selection methods selected different features, relatively consistent classification results were obtained under different classifiers, among which Lasso feature selection method achieved the best classification results. When using datasets 1–3 for modeling, the results on the testing set were poor, with AUC values ranging from 0.54 to 0.64. According to the definition of AUC value, in the range of [0.5–0.7], although the model has certain prediction ability, its prediction level is relatively poor. The AUC values of 0.76, 0.78, and 0.79 in the testing set of the merged dataset were obtained respectively, showing good predictive performance.

For further analysis, we evaluated the accuracy, sensitivity, specificity and AUC values of different models. The results are shown in **Table 3**. When modeling with dataset 1, the average accuracy, sensitivity, specificity, and AUC values on dataset 2 were 0.45, 0.73, 0.36, and 0.58, respectively; the average accuracy, sensitivity, specificity, and AUC values on dataset 3 were 0.64, 0.70, 0.43, and 0.59, respectively. When modeling with dataset 2, the average accuracy, sensitivity, specificity, and AUC values on dataset 1 were 0.48, 0.40, 0.69, and 0.57, respectively; the average accuracy, sensitivity, specificity, and AUC values on dataset 3 were 0.46, 0.41, 0.67, and 0.56, respectively. When modeling with dataset 3, the average accuracy, sensitivity, specificity and AUC values on dataset 1 were 0.62, 0.72, 0.38, and 0.58, respectively; the average accuracy, sensitivity, specificity, and AUC values on dataset 2 were 0.47, 0.69, 0.40, and 0.58, respectively. When modeling with the merged dataset, the average accuracy, sensitivity, specificity and AUC values on the testing set were 0.74, 0.77, 0.68, and 0.78, respectively. Based on the above results, it can be seen that the classification results are the best when using the merged dataset for modeling.

**Table 3:** Classification results of different testing sets.

Training set	Testing set	Accuracy	Sensitivity	Specificity	AUC
Dataset 1	Dataset 2	0.45	0.73	0.36	0.58
	Dataset 3	0.64	0.70	0.43	0.59
Dataset 2	Dataset 1	0.48	0.40	0.69	0.57
	Dataset 3	0.46	0.41	0.67	0.56
Dataset 3	Dataset 1	0.62	0.72	0.38	0.58
	Dataset 2	0.47	0.69	0.40	0.58
Merged training set	Merged testing set	0.74	0.77	0.68	0.78

## Discussion

This paper studied the subtype differentiation of NSCLC based on radiomics analysis. The identification of histological types of NSCLC is essential for treatment, and many studies have been conducted and demonstrated the potential ability of radiomics. However, existing studies usually focus on relatively small datasets and lack multi-center external validation datasets, resulting in a high risk of over-fitting, so the model’s generalization performance cannot be adequately verified. Besides, the methods used in many studies are relatively simple, and few feature selection and classifier methods are compared. The differences between different methods are not fully discussed, which reduces the credibility of the model and limits its clinical application.

To solve the above problems, we retrospectively studied three independent datasets from different centers, where each dataset was used to train the model and tested in the remaining two datasets. Then all the datasets were combined into a large dataset and randomly divided into training and testing sets for modeling and analysis. For each form of dataset division, a corresponding radiomics model was constructed. The experimental results show that each model’s performance is quite different, and the model based on the merged dataset obtains the best performance.

The feature subsets selected by different feature selection methods vary greatly, which is also the difficulty of radiology research. How to select the most effective feature subset is a complex feature engineering problem, especially in radiomics research. Besides, how to ensure the interpretability of features is another difficulty in applying radiomics models to the clinic. In future work, we will continue to conduct research in this field by combining doctors’ qualitative semantic features and deep learning features, and using ensemble methods to select interpretable and distinguishable features.

Generalization ability is an important index in radiomics research. The samples we studied came from different centers, with different imaging methods and a wide range of demographic information. The experimental results show that the performance of the other centers’ datasets was poor when only one dataset was used for modeling. The results put forward a requirement for future radiomics research; that is, to better apply it to clinical practice, it is necessary to collect as much multi-center datasets as possible in order to learn the common feature representation. When using multi-center datasets, the imaging quality is another issue that needs to pay attention. Some notable works have discussed imaging quality issues (28–30), which inspire us to carry out future work.

The curse of dimensionality is a huge challenge in the radiomics analysis, so feature selection is an essential step. Many studies have discussed the performance of different feature selection methods. Qian et al. (31) evaluated 12 feature selectionmethods and 7 classification methods to distinguish glioblastoma from solitary brain metastases and found that the Lasso feature selection and SVM classifier obtained the highest AUC. Wu et al. (11) investigated 24 filter-based feature selection methods and 3 classification methods for the classification of lung cancer histology, and found that the ReliefF feature selection method has higher prediction accuracy than other methods. In this study, we studied three widely used feature selection methods, namely mRMR, Lasso and SFS. The experimental results demonstrated that although different feature selection methods selected different features, relatively consistent classification results were obtained under different classifiers, among which Lasso feature selection method achieved the best classification results.

Since the samples we studied came from different institutions, the process of tumor segmentation by different radiologists and the repeatability of radiomic features may vary significantly. Subjectivity may occur in the determination of tumor volume and tumor boundaries, leading to uncertainties of tumor segmentation, which may adversely affect the repeatability of radiomic features. It is widely acknowledged that it is difficult to precisely delineate the tumor volume due to the similar characteristics between organs and tumors, as well as the differences in shape and size of the tumor. Moreover, medical images are far from perfect because they have limited resolution and may contain artifacts. Physicians often interpret tumors differently based on their skills and experiences. Since radiomic features are calculated based on the tumor masks, the uncertainties of the tumor segmentation significantly affect the features, resulting in poor generalization performance of the prediction models (32). With the development of computer vision and deep learning, automatic tumor segmentation may help radiomics studies. One of our recent work shows that the classification results based on automated segmentation and ground truth segmentation have no significantdifferences in computer-aided glioma grading task (33). In the future work, we will combine the automatic segmentation method for radiomics research.

## Data Availability Statement

The original contributions presented in the study are included in the article/supplementary material, further inquiries can be directed to the corresponding authors. 

## Ethics Statement

The studies involving human participants were reviewed and approved by The Ethics Committee of Shandong Cancer Hospital. The patients/participants provided their written informed consent to participate in this study. Written informed consent was obtained from the individual(s) for the publication of any potentially identifiable images or data included in this article.

## Author Contributions

FY and WC collected and analyzed the data, wrote the initial draft, and accomplished the final version. HW and XZ analyzed the data. SY, XQ, and YC conceptualized, designed, and supervised the study, and revised the article. All authors contributed to the article and approved the submitted version. 

## Funding

This work was supported by the National Natural Science Foundation of China (Grant nos. 81872475, 81372413, and U1806202), the Department of Science and Technology of Shandong Province (2017CXGC1502), and the Taishan Scholars Project in Shandong Province (ts201511106).

## Conflict of Interest

The authors declare that the research was conducted in the absence of any commercial or financial relationships that could be construed as a potential conflict of interest.
